# Multi-resBind: a residual network-based multi-label classifier for in vivo RNA binding prediction and preference visualization

**DOI:** 10.1186/s12859-021-04430-y

**Published:** 2021-11-15

**Authors:** Shitao Zhao, Michiaki Hamada

**Affiliations:** 1 Waseda Research Institute for Science and Engineering, Waseda University, 3-4-1 Okubo Shinjuku-ku, Tokyo, 169-8555 Japan; 2 Department of Electrical Engineering and Bioscience, Faculty of Science and Engineering, Waseda University, 3-4-1 Okubo Shinjuku-ku, Tokyo, 169-8555 Japan; 3 Computational Bio Big-Data Open Innovation Laboratory (CBBD-OIL), National Institute of Advanced Industrial Science and Technology, 3-4-1 Okubo Shinjuku-ku, Tokyo, 169-8555 Japan; 4 Graduate School of Medicine, Nippon Medical School, 1-1-5 Sendagi, Bunkyo-ku, Tokyo, 113-8602 Japan

**Keywords:** RNA-binding protein, Residual network, Multi-label classification, Integrated gradients, Photoactivatable ribonucleoside enhanced cross-linking and immunoprecipitation

## Abstract

**Background:**

Protein-RNA interactions play key roles in many processes regulating gene expression. To understand the underlying binding preference, ultraviolet cross-linking and immunoprecipitation (CLIP)-based methods have been used to identify the binding sites for hundreds of RNA-binding proteins (RBPs) in vivo. Using these large-scale experimental data to infer RNA binding preference and predict missing binding sites has become a great challenge. Some existing deep-learning models have demonstrated high prediction accuracy for individual RBPs. However, it remains difficult to avoid significant bias due to the experimental protocol. The DeepRiPe method was recently developed to solve this problem via introducing multi-task or multi-label learning into this field. However, this method has not reached an ideal level of prediction power due to the weak neural network architecture.

**Results:**

Compared to the DeepRiPe approach, our Multi-resBind method demonstrated substantial improvements using the same large-scale PAR-CLIP dataset with respect to an increase in the area under the receiver operating characteristic curve and average precision. We conducted extensive experiments to evaluate the impact of various types of input data on the final prediction accuracy. The same approach was used to evaluate the effect of loss functions. Finally, a modified integrated gradient was employed to generate attribution maps. The patterns disentangled from relative contributions according to context offer biological insights into the underlying mechanism of protein-RNA interactions.

**Conclusions:**

Here, we propose Multi-resBind as a new multi-label deep-learning approach to infer protein-RNA binding preferences and predict novel interactions. The results clearly demonstrate that Multi-resBind is a promising tool to predict unknown binding sites in vivo and gain biology insights into why the neural network makes a given prediction.

**Supplementary Information:**

The online version contains supplementary material available at 10.1186/s12859-021-04430-y.

## Background

RNA-binding proteins (RBPs) regulate gene expression through specific protein-RNA interactions that play key roles in various post-transcriptional gene regulation processes [1, 2]. RBPs interact with RNAs and regulate their functions via one or several RNA-binding domains (RBDs). The canonical functional domains include the RNA recognition motif [3], K homology [3], C3H1 zinc finger [4], and Piwi/Argonaute/Zwille domain [5]. The RNA specificity is considered to be largely determined by the sequence of a given RBP or RBD, as well as the structural context [6].

RNAcompete is a microarray-based high-throughput method for in vitro RBP binding preference determination [7], in which purified epitope-tagged RBPs of interest are used to screen RNA sequences of 38–41 nucleotides from a designed RNA pool. In the pool, sampling without replacement occurs at least 16 times among all possible combinations of 9-mers. The bound RNAs are then identified through hybridization with DNA strands, resulting in the relative affinity of a specific RBP against more than 240,000 short RNAs. RNAcompete-2013, a benchmark dataset for RBP prediction, expanded the number of RBPs to 244 across many RBP families [8]. The combined ultraviolet cross-linking and immunoprecipitation with sequencing (CLIP-seq) method was developed to measure genome-wide protein-RNA interactions in different cellular environments [9–12]. CLIP-seq and its variants usually provide a large number of target sites through reads mapping. However, owing to the high level of noise or non-specific background, it is often difficult to find specific binding motifs using these data. In particular, RNase T1 enzyme-inducing sequence bias is a common limitation in many CLIP-seq experiments [13].

Although these advanced experimental methods (RNAcompete and CLIP-seq) have made it possible to use computational approaches to predict protein-RNA interactions, they require sifting through huge amounts of potential binding sites. The prediction problem can be defined as determining the binding preference of a given RBP from a large number of short or long RNA sequences. Initially, the binding specificity of a given RBP is defined as a motif discovery problem using only the sequence information. Traditional prediction methods include a position weight matrix (PWM) and hidden Markov models such as MEME [14] and MatrixREDUCE [15]. MEMERIS [16] was the first computational tool developed to predict binding sites by integrating RNA accessibility information. Moreover, RNAcontext [17] and RCK [18] were developed to extend the RNA structural profile from simply paired or unpaired to five structural annotations as follows: paired, hairpin, inner, multi, and external (i.e., PHIME). GraphProt [19] utilizes a graph kernel to extract binding preferences based on structure information, which was successfully applied to in vivo CLIP-seq datasets.

As the first application of deep learning for the prediction of protein-nucleic acid interactions, DeepBind [20] surpassed the state-of-the-art model (PWM/k-mer) across a wide collection of datasets both in vitro using protein binding microarrays and in vivo using chromatin immunoprecipitation-sequencing data. Two convolutional neural network (CNN)-based methods, cDeepBind [21] and DLPRB [22], were also developed, which integrate RNA structure annotations (PHIME) into the model, and exhibited substantial improvement over both DeepBind and RCK. Subsequently, ThermoNet [23] was proposed, which integrates a k-mer embedding CNN model to RNA sequences with an ensemble of secondary structures, and exceeded the prediction accuracy of cDeepBind and DLPRB. By adapting the residual network (ResNet) [24] to protein-RNA interaction prediction, which is very successful in Computer Vision, ResidualBind [25] achieved state-of-the-art performance in RNAcompete 2013. Another ResNet-based method, PrismNet [26], accurately extracts sequence/structure binding preferences of in vivo protein-RNA interactions through the use of novel structural annotation of icSHAPE reactivity scores [27]. Similarly, RPI-Net [28] and GraphProt2 [29] utilize graph neural networks via encoding input sequences as graphs to predict protein-RNA interactions in CLIP-seq datasets. Compared with GraphProt and CNN-based methods, both RPI-Net and GraphProt2 offer substantial improvements for a wide variety of individual CLIP-seq datasets. Despite these improvements, all of these models learn from an individual RBP dataset, and thus commonly suffer from experimental bias (GC-bias in RNAcompete 2013 and RNase T1 enzyme-inducing sequence bias in CLIP-seq [13]). DeepRiPe [30] utilizes a multi-task module to predict the binding sites of multiple RBPs simultaneously, thereby suppressing the sequence bias introduced by the experimental protocol. MultiRBP [31] applies the multi-task or multi-label learning to RNAcompete dataset and also obtained promising results compared to training a single model per RBP. However, the prediction accuracy of DeepRiPe is limited due to the shallow neural network architecture. DeepRiPe also cannot determine the sequence motif and the binding pattern of other features simultaneously owing to the use of independent convolution kernels in multimodal learning.

To solve these limitations of current binding prediction methods, we developed Multi-resBind, a multi-label deep-learning approach for protein-RNA binding prediction. We evaluated the performance of Multi-resBind in terms of the area under the receiver operating characteristic curve (AUROC) and average precision (AP) relative to those of DeepRiPe using the same large-scale PAR-CLIP datasets. Extensive studies were further conducted on a series of various types of input data and their combinations. We also explored the problem of imbalanced data across classification classes in multi-label learning, which is common when dealing with biological data. Finally, we used modified integrated gradients (IG) [32] to generate a series of attribution maps to evaluate the performance of Multi-resBind through visual inspection.

## Methods

### Input dataset

The input dataset was a collection of preprocessed PAR-CLIP data for 59 RBPs in the HEK293 cell line as in a previous study [30]. Preprocessing of raw data was performed using the same pipeline in [33], including PARalyzer [34] for peak calling. During reads mapping, an older version of the reference human genome (GRCh37/hg19) was selected for annotation. The dataset was split into three categories: low, medium, and high with peaks of <15,000, >15,000 but <100,000, and >100,000, respectively. In each category, 70% of the data consisting of RNA sequences and region types were used for training the model, and 20% and 10% of the data were treated as validation and test datasets, respectively.

In the original datasets downloaded from the Ohler Laboratory website, there are 150-nucleotide RNA sequences with 250-nucleotide region type annotations. To equalize the lengths of the input of region types and RNA sequences, we reduced the region types from 250 to 150 bases at the same position. RNA sequences with one nucleotide (A, U, G, or C) and region types with one annotation (3′ UTR, 5′ UTR, CDS, or intron) in each position were converted to a one-hot format, respectively. RNA sequences with a length of 150 bases in the whole dataset were converted into the fasta format and then employed as the input to calculate two types of structural profiles. Structural profiles with paired-unpaired annotations were calculated using a modified script of RNAplfold [35], whereas profiles with a stem (S), hairpin (H), bulge (B), internal (I), multibranch (M), or exterior (E) loop were calculated using CapR [36]. Readily available data were concatenated along the features axis in the same position, resulting in a 150 × *d* two-dimensional matrix to feed the first convolutional layer.

For the input data of eCLIP experiments, we firstly downloaded the processed bed files from ENCODE Project (https://www.encodeproject.org). The reliable peaks were filtered using a strict cutoff value (fold change > 3 and *p*-value < 0.001) and merged between two replicates for each RBP. RBPs with less than 1000 reported peaks were removed from the datasets. The eCLIP datasets were then divided into five categories for each cell line: low, medium 1, medium 2, high 1 and high 2 with peaks of >1000 but <2000, >2000 but <4000, >4000 but <7000, >7000 but <10,000 and >10,000, respectively. The bin sizes for spliting the genome were extended to 100bp from 50bp to account for the eCLIP peaks resolutions. Other steps were utilized the same approach as PAR-CLIP datasets.

### Neural network architecture

We designed Multi-resBind as a multi-label classifier taking sequence data with inherent features (nucleotides, region types, and structure profiles) as input to give *k* prediction probabilities (range from 0 to 1) of the corresponding category for RBPs of interest as the output. Compared with DeepRiPe, we used the ResNet block to increase the number of convolutional layers of the neural network to enable the learning of more expressive feature maps from the high-noise CLIP-seq dataset, and treated different types of input data as a feature of the time-series data to allow for the simultaneous determination of more complex patterns from the given context (sequence and structure or sequence and region) that is important for protein-RNA binding.

**Fig. 1:**
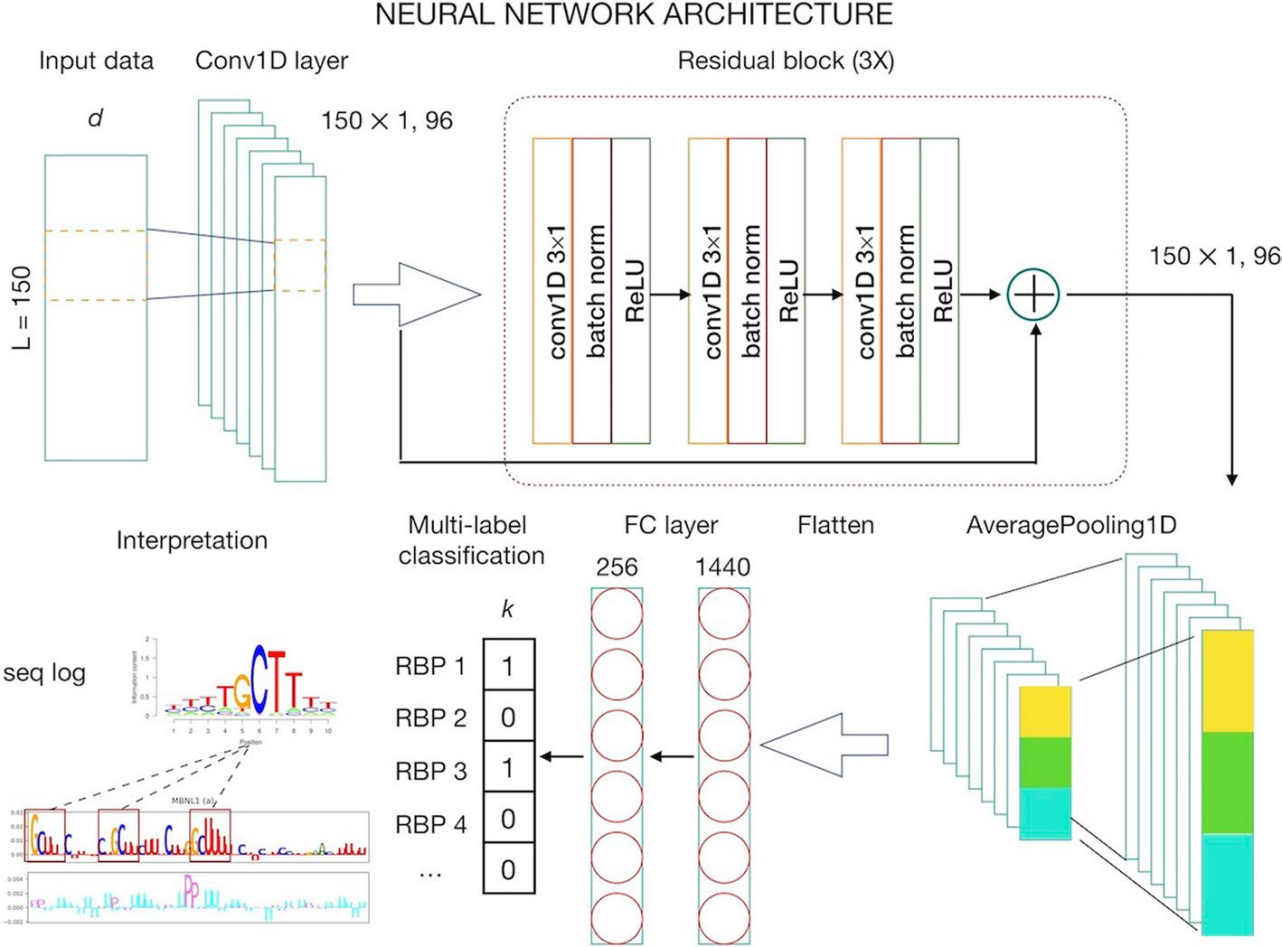
Simplified diagram of the neural network of the Multi-resBind model. The input data with a length of 150 and width of *d* is first fed to a 1D convolutional layer. In this layer, the number of kernel filters, kernel size, and step size of the stride are set to 96, $11 \times d$, and 1, respectively. The obtained feature maps are provided as input to a residual block (3 ×) with skip connection in the last block. Each block consists of three sequential layers: convolution 1D, batch normalization, and ReLU activation. After the residual block, the average pooling with 10 × 1 receptive fields (size = 10, stride = 10) and concatenate operations convert the feature maps to a 1D vector. The 1D vector then passes through a 3-layer fully connected network with 256 nodes in the hidden layer. The last fully connected layer consists of *k* nodes corresponding to the RBPs of interest independently. Finally, sigmoid is chosen as the activation function of each node in the last layer

Multi-resBind consists of four parts. The first part of the model is a 1D convolutional layer for the sequence data in which the number of filters was set to 96 and the filter (kernel) size was set to 11. The second part of the model is a 3× residual block. Each residual block consists of three layers, including convolution 1D, batch normalization, and activation (ReLU). The dropout probability in each residual block was set to 0.1, 0.2, and 0.5, respectively. At the end of this part, a skipped connection was added to integrate the output of the 3× residual block and 1D convolutional layer. The third part of the model is the AveragePooling1D layer with pool size of 10 and strides of 10 using the *valid* padding option and the fully connected layer (256 hidden nodes). The fourth part of the model is *k* independent nodes with a sigmoid activation function producing prediction probabilities of 0–1. Figure 1 shows a simplified diagram of the network architecture of the Multi-resBind model.

### Training procedure

For each dataset in the three categories (low, medium, and high), we trained a unified model with *k* separated nodes by minimizing the mean value of binary cross-entropy (BCE) between the predicted probabilities of each node and assigned labels (0 or 1) across *k* classes per sample. The model was trained with a mini-batch (the default batch size was set to 128) stochastic gradient descent method called Adam [37] to update the weights in the neural networks. The initial learning rate was set using a default value of 0.001 and training epochs were set to 40. At the end of each epoch, the model is saved when training loss reaches the minimum value for the validation set. At the beginning of model training, the initialization layer weights of each model are determined with a Glorot initializer (also known as Xavier) [38].

### Evaluation scores

After the model was trained on the training and validation datasets, its performance was evaluated using an independent hold-out test dataset. The classification performance for each RBP was assessed through two indicators for binary classification evaluation: AUROC and AP. The AUROC is the most commonly used indicator for evaluating the performance of a binary classification model with balanced positive and negative samples. AP is defined as the area under the precision-recall curve, which measures the trade-off between precision and recall at different decision thresholds. In the context of multi-label classification, AP is more distinctive than AUROC when there are more negative than positive labels per class. Therefore, the model with a higher AP score is considered to perform better in higher-ranked samples, where more positive samples are classified correctly [39].

### Model interpretability

The attribution maps were generated using IG, an advanced gradient-based method [32]. The core concept of this method is similar to that of DeepLIFT [40], which was used to solve the gradient saturation problem in the interpretability of the “black box” model. When the input *X* with *i*th dimension features (nucleotide, region, and structure profile), predicted output F(*X*), and baseline point *X*′ are known, IG is defined as follows:

$$\begin{aligned} IG(X , X^{\prime} ) {{:}{:}{=}} (X -X^{\prime})*\int _{0}^{1}\nabla {F}(\alpha *X +(1-\alpha )*X^{\prime} )\mathrm {d}\alpha \end{aligned}$$

where $\partial {F(X)}/\partial {X_i}$ is the gradient of *F* along the *i*th dimension at *X*. In practice, we approximated the path integral by linear interpolation (with a default value of 50 steps). Choosing the baseline or reference point is another challenging problem, and is usually based on the specific application. According to previous studies [41, 42], we did not choose zero as the reference point; instead, the reference point was generated by taking the average of 2000 samples with the lowest predicted score for the RBP of interest.

## Results

### Multi-resBind achieves state-of-the-art performance using the PAR-CLIP dataset

**Fig. 2:**
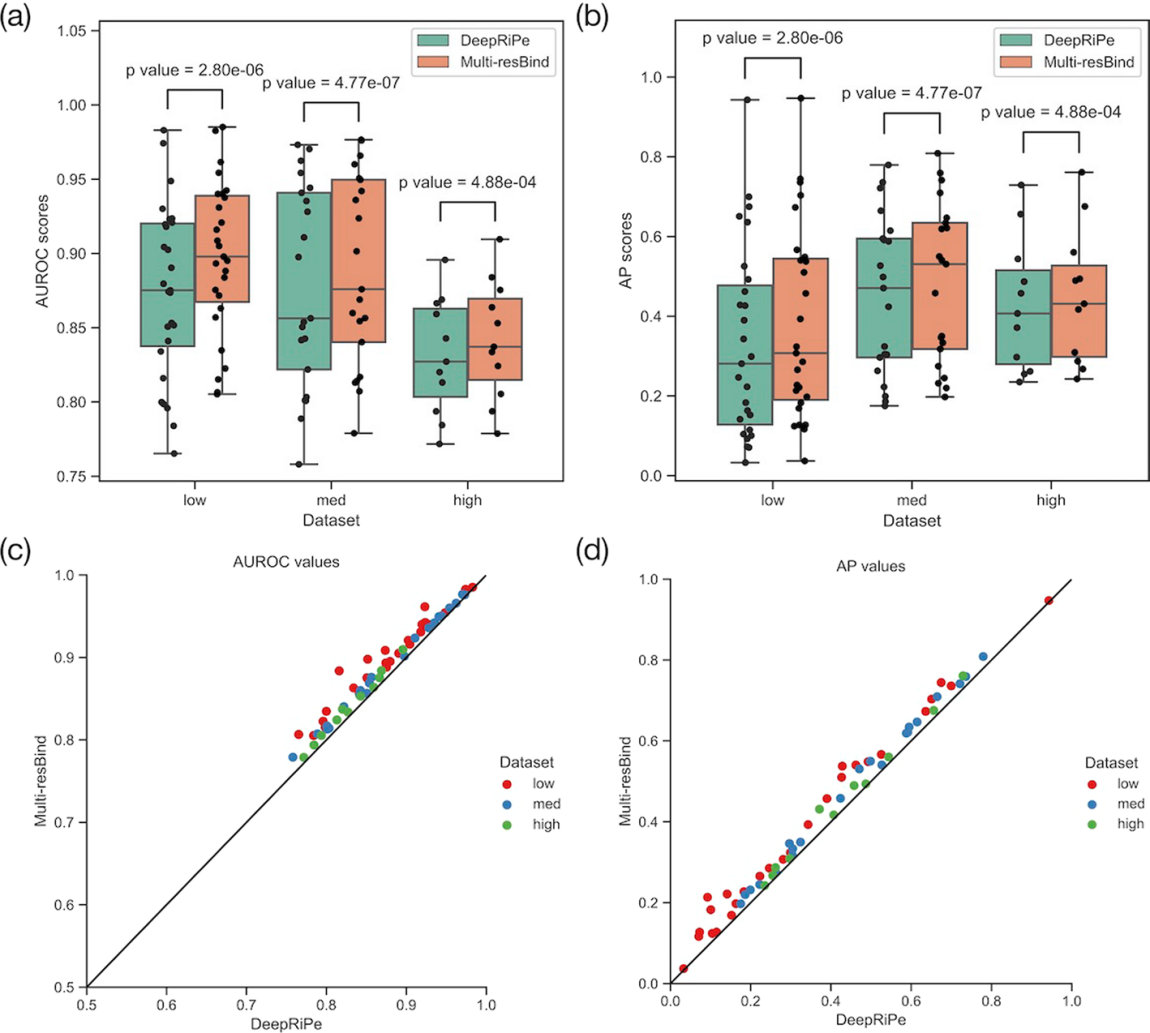
Performance comparison between Multi-resBind and DeepRiPe utilizing RNA sequences and region types as input. Results are based on three different dataset types [low, medium (med), and high]. The low dataset consists of 27 RBPs with binding peaks less than 15,000, the med dataset consists of 21 RBPs with binding peaks greater than 15,000 and less than 100,000, and the high dataset consists of 11 RBPs with binding peaks greater than 100,000. In the boxplot, the y-axis represents the evaluation index (AUROC or AP scores) and the x-axis represents different datasets. The two methods are distinguished by color coding. The one-sided Wilcoxon signed-rank test was employed to calculate the *p* values. In the scatterplot for pairwise comparison, the y-axis represents the evaluation index (AUROC or AP scores) for Multi-resBind and the x-axis represents the evaluation index (AUROC or AP scores) for DeepRiPe of the same RBP. Different datasets are distinguished by color coding. **a** Boxplots for the performance comparison of Multi-resBind and DeepRiPe based on AUROC scores. **b** Boxplots for the performance comparison of Multi-resBind and DeepRiPe based on AP scores. **c** Scatterplot for the pairwise comparison of Multi-resBind and DeepRiPe based on AUROC scores. **d** Scatterplot for the pairwise comparison of Multi-resBind and DeepRiPe based on AP scores

To compare Multi-resBind against DeepRiPe, we employed the same input data (sequence and region information) and weighted loss function for prediction analysis from the PAR-CLIP dataset. As shown in Fig. 2a, b, Multi-resBind showed significant improvement over DeepRiPe with respect to both the AUROC and AP scores across the three categories. Although some region information was lost (due to reducing the length from 250 to 150 nucleotides) to equalize to the length of input sequences, our method still outperformed DeepRiPe for each RBP, as shown in Fig. 2c, d. These results suggested that using ResNet with the skipped connection to build deeper networks could be a more potent approach to solve prediction problems in related biological research fields.

To demonstrate the generality of Multi-resBind model, we further conducted comparision experiments on two eCLIP datasets (cell lines of K562 and HepG2). The preparation process of the entire dataset refers to the description in the DeepRiPe’s paper or the method section. The performance of the models in terms of mean AUROC and AP are provided in Additional file 1: Table S1. In short, the results of the additional experiments are consistent with our results (see Fig.2 and Additional file 2: Files S1) and main conclusion on DeepRiPe and Multi-resBind models.

To demonstrate the usefulness of each module, we also did the ablation experiments cross three PAR-CLIP datasets (low, med and high) for Resnet block and pooling layer, and summarized the results into Additional file 1: Table S2. The data in Additional file 1: Table S2 shows the addition of Resnet block has a significant improvement in performance on two evaluation indicators of mean AUROC and mean AP. We can also see that there is no obvious difference between max pooling layer and average pooling layer. The performance of average pooling has a slight advantage over max pooling on the dataset (low).

**Fig. 3:**
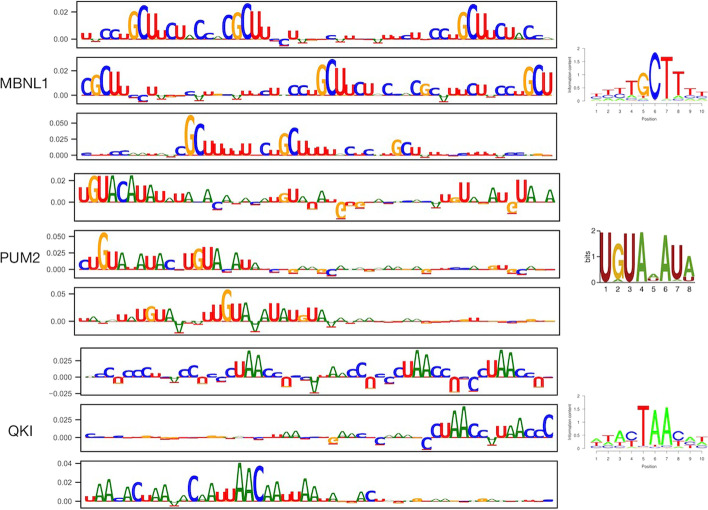
Attribution maps of several respective RBPs (MBNL1, PUM2, and QKI), utilizing the sequence contribution only. For each RBP, the three sequences with the highest prediction scores were used to calculate gradients and generate the corresponding contribution relative to the predicted probability. The y-axis represents the relative contribution of each base and the x-axis represents the corresponding nucleic acid (A, U, G, or C) in the RNA sequence. Attribution maps for model interpretation were constructed using the IG method, in which the reference point was generated by taking the average of 2000 samples with the lowest prediction score. The sequence logos corresponding to attribution maps were generated through the RCAS profiles of the RBP binding sites [33]. The sequence logo of PUM2 was obtained from a pulished study [10]

### Effects of different input data types and their combination

RNA structure is an important feature for RBP recognition along with the sequence. Many existing models, including RCK, RNAcontext, DRPLB, cDeepbind, and ThermoNet, have demonstrated the relevance of structural information using an in vitro dataset (RNAcompete). However, Koo et al. demonstrated that RNA secondary structure profiles do not increase a model’s performance using RNAcompete [25]. Recently, Sun et al.’s study shown that RNA secondary structure is dependent on cellular environment. This difference is especially important in regulating dynamic protein-RNA bindings across various conditions (e.g K562 and HepG2) [26]. Hence, it is also necessary to compare the influence of different types of input data on the prediction of RBP interactions in vivo.

All calculation results are summarized in Table 1 and Additional file 1: Fig. S1. All predict results (AUROC and AP) per RBP using RNA second structure profiles (RNAplfold) are saved in Additional file 3: Files S2 and Additional file 4: Files S3. When we input the sequence, secondary structure, and region information separately, the performance of the model decreased sequentially (i.e., RNA sequence > region types > secondary structure). Thus, sequence information was the most important feature for prediction. When combining data in pairs (sequence_structure, sequence_region, and structure_region) as input, the combination of sequence and region showed the best performance. The performance of this combination also surpassed that of the model that considered all three components (sequence, structure, and region) as input. This result is mainly considered to reflect the relatively low prediction accuracy of RNA secondary structure in vivo via computational tools such as RNAfold [43]. Using different structural profiles (SHBIME) as the input slightly improved the predictive performance. The computational results using structural profiles predicted by CapR are summarized in Additional file 1: Table S3, Additional file 5: Files S4 and Additional file 6: Files S5.

**Table 1:** Performance of the Multi-resBind model with various types of input data

Data types	Data dimensions	Mean AUROC	Mean AP
Sequence	(150,4)	0.8809	0.3372
Structure	(150,2)	0.6987	0.1189
Region	(150,4)	0.6710	0.0912
Sequence and structure	(150,6)	0.8843	0.3521
Sequence and region	(150,8)	**0.8976**	**0.3808**
Structure and region	(150,6)	0.7602	0.1602
Sequence, structure and region	(150,10)	0.8957	0.3714

Evaluation experiments were performed using different input features and their combinations with a held-out test set in the 27 RBPs low dataset. Among the features, sequence represents one-hot encoded nucleic acid bases (A, U, G, or C), structure represents paired or unpaired structure profiles predicted by a modified script of RNAplfold, and region represents one-hot encoded region type information (3′ UTR, 5′ UTR, CDS, or intron) of the corresponding sequence. The mean AUROC and the mean AP refer to the average AUROC and AP scores of the 27 RBPs in the low dataset. The numbers marked in bold represent the maximum value under the given evaluation metric

### Cost-sensitive multi-label learning does not contribute to RBP prediction

We encountered a problem of data imbalance in the RBP prediction task in which instances for different classes were not represented equally (Additional file 1: Fig. S2), leading to a bias toward the prediction of the more frequent classes. The difference in the positive label count between each class was extremely large. The experimental results showed that the performance (based on AP) of the trained neural network (the original DeepRiPe model) had a strong positive correlation with the number of positive labels in each class (Additional file 1: Fig. S3). Therefore, we comprehensively compared a series of different loss functions using a cost-sensitive strategy to improve the performance of predictive models. Compared with BCE, all methods tested resulted in limited improvement (Additional file 1: Table S4). Comprehensive consideration of performance on the two indicators (AUROC and AP scores) established that BCE was still the best choice. All predict results (AUROC and AP) per RBP using different loss functions under the Multi-resBind model are saved in Additional file 7: Files S6 and Additional file 8: Files S7.

### Attribution maps highlight important sequence motifs

The interpretation of predictive models has attracted increasing attention in biological research, especially to determine whether the underlying learning mechanism of a model is consistent with current biological knowledge. In the case of deep learning, the generation of an attribution map for a particular RBP, and evaluating its consistency with sequence motifs based on k-mers and PWMs have become mainstream practice. Toward this end, we generated attribution maps for positive samples using the test data (predicted scores > 0.5 and labels = 1). Sequences and the corresponding regions with the three highest prediction scores were selected as input for constructing the maps. Figure 3 shows the attribution maps of sequences for several target RBPs exhibiting high performance (AP > 0.50) using the low dataset, which were generated using an average value of 2000 samples with the lowest predicted scores as a reference point. Other attribution maps using the zero vector as a reference point are shown in Additional file 1: Fig. S4.

We further conducted a case study and compared the attribution maps to published known motifs. As an example, MBNL1 is a splicing factor with reported YGCU/GCUU binding motifs, in which Y refers to C or U, and its attribution map was found to include repeated motifs in the mRNAs. In the attribution map, QKI was also highlighted owing to its reported ACUAAY binding motifs. A much longer well-established UGUAHAUA binding motif for PUM2 was also observed in the attribution map with a simple pattern, in which H refers to A, C, or U. The Multi-resBind model could even learn the subsequence of a canonical binding motif or a major part of the motif containing one or two mismatches at any position.

### Attribution maps reveal region and structure preferences for RBP prediction

As shown in Table 1, the combination of sequence and region or structural information significantly improved the predictive power of the Multi-resBind model compared to only using sequence information. The combination pattern (sequence and region or sequence and structure) observed on the attribution map further facilitated interpretation of protein-RNA binding.

**Fig. 4:**
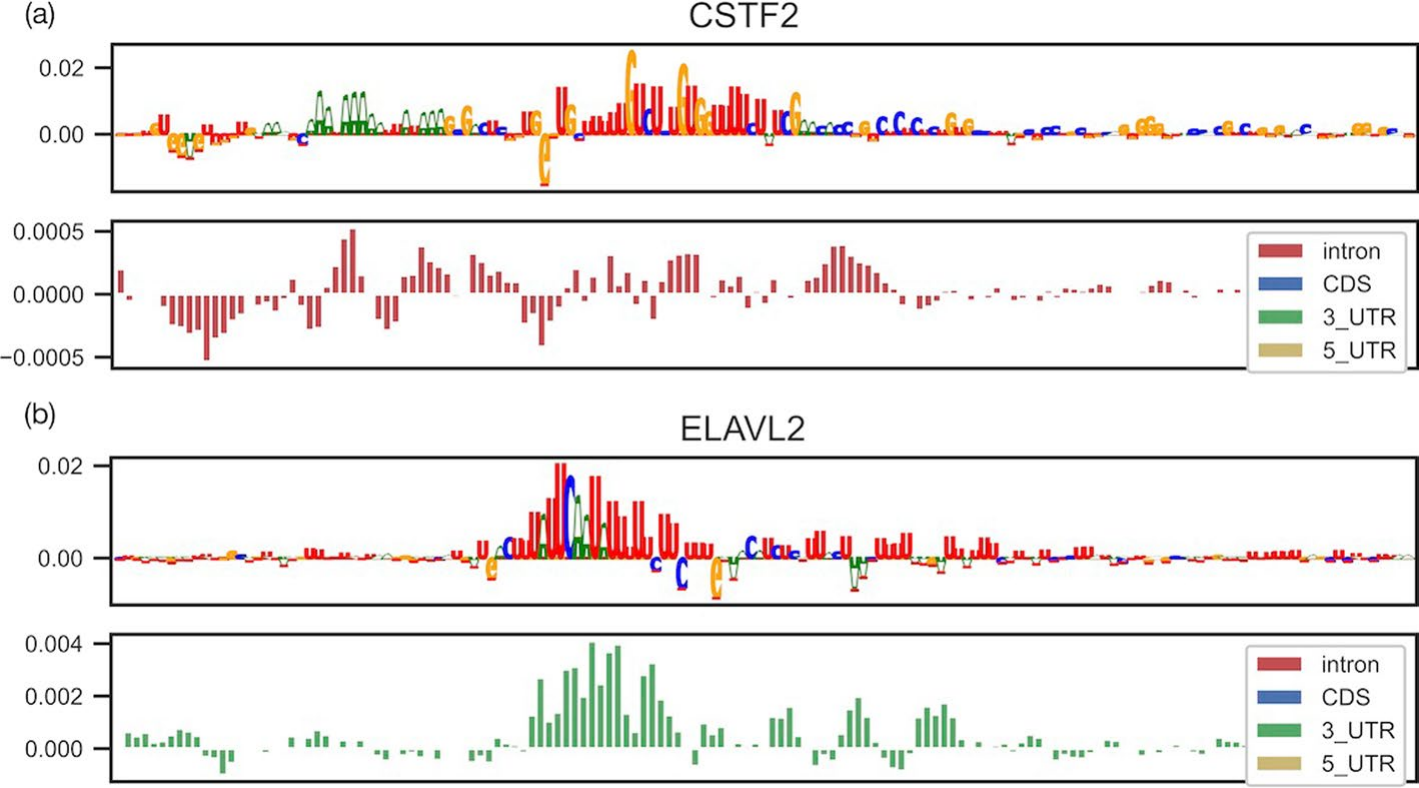
Attribution maps for RBPs of interest (ELAVL2 and CSTF2) with sequences and regional context. Each subgraph (**a**) or (**b**) consists of two boxplots (top and bottom). In the upper graphs, the y-axis represents the relative contribution of each base and the x-axis represents the corresponding nucleic acid base (A, U, G, or C) in the RNA sequence. In the lower graphs, the y-axis represents the relative contribution of each base and the x-axis assigns one box with a different color for each category of the corresponding region type annotation (3′ UTR, 5′ UTR, CDS, or intron) in the RNA sequence. The attribution maps were generated through a trained model using sequences and region as input

For example, ELAVL2 regulates mRNA stability and translation via binding to U-rich elements in the 3′ UTR of target mRNAs. In Fig. 4a, the highlighted U-rich elements in sequences with specific region preferences of the 3′ UTR are in agreement with previous studies [44]. CSTF2 (also known as CstF-64) interacts directly with distal sequence elements (GU-rich regions) [45, 46] as a member of the cleavage stimulation factor complex, which is involved in the 3′-end cleavage and polyadenylation of pre-mRNAs. Figure 4b shows that CSTF2 specifically recognizes the U/GU-rich elements located downstream of the AAUAAA sequence required for cleavage [47]. The region preferences of the intron were also confirmed in a previous study demonstrating that almost half of all CstF64 binding sites are located in introns, and these intronic CstF64 binding sites are more strongly conserved than randomly located intronic sequences [48].

**Fig. 5:**
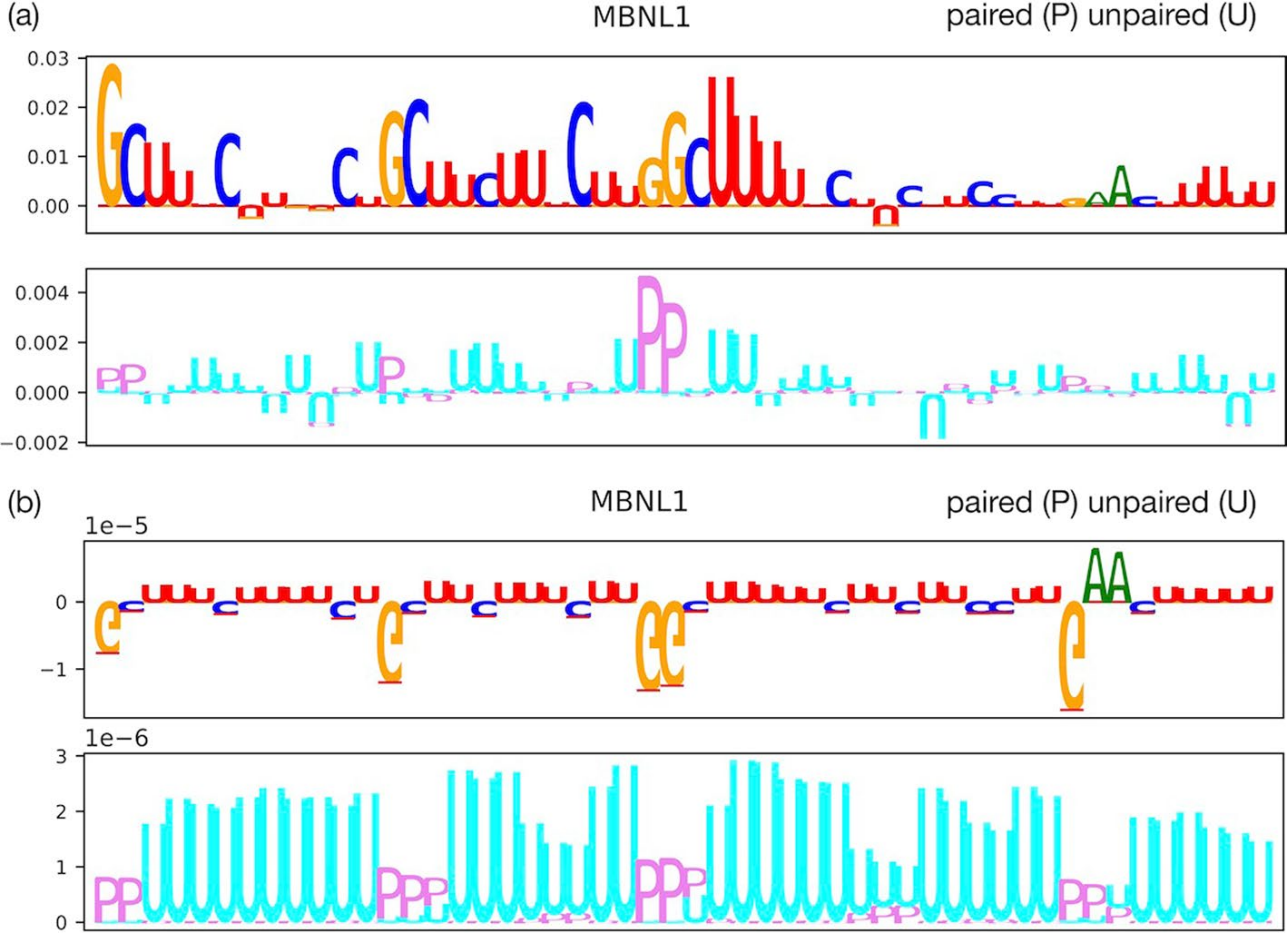
Attribution maps for MBNL1 with sequence and structure profiles as the input. In the upper graphs of (**a**) and (**b**), the y-axis represents the relative contribution of each base and the x-axis represents the corresponding nucleic acid base (A, U, G, or C) in the RNA sequence. In the bottom graphs of (**a**) and (**b**), the y-axis represents the relative contribution of each base and the x-axis represents the corresponding structure annotation (paired or unpaired) in the RNA sequence. Maps in (**a**) were generated by taking the average of 2000 samples with the lowest prediction score as the reference point, whereas maps in (**b**) were generated using the zero vector as the reference point

It remains unclear whether MBNL1 mainly recognizes single- or double-stranded RNA elements. Lambert et al. indicated that MBNL1 binding can tolerate GC pairing, but largely favors unpaired U bases [49]. We generated two attribution maps with the highest prediction scores of MBNL1 using the sequences and structure information (PU) as input. As shown in Fig. 5a, unpaired structures (violet) are consistently highlighted under the GCs in the attribution map, whereas paired structures (cyan) are consistently highlighted under the U bases.

## Discussion

With development and performance evaluation of Multi-resBind, we have shown that careful design of a CNN-based deeper network architecture could significantly improve the prediction power for RBP binding sites. Other deeper backbone modules such as Xception [50] are available that have not yet been introduced into this research field. In addition, Transformer [51], ELMo [52], and BERT [53], which have shown great success in the field of natural language processing, also have beneficial properties for processing biological sequences.

The influence of different types of input data on prediction performance can be considered from the perspective of feature engineering. For processing different types of source data, many models adopt multimodal forms, such as DeepRiPe [30]. With Multi-resBind, we processed sequence data more traditionally as time-series data, considering sequence, structure, and region type information as different features for each nucleic acid (time step). The experimental results further proved that this approach has some advantages in network architecture design and binding motif mining. For in vivo prediction, a recent study showed that the structure profile predicted by calculation software does not contribute to improving the prediction performance of a deep-learning model, because the structure of mRNAs differs among cell lines. The icSHAPE data [27] is a very promising supplement information for RNA secondary structure or protein-RNA binding prediction [26].

We also investigated the impact of the loss function when dealing with data imbalance on prediction performance without changing the network architecture. For models designed in an end-to-end manner, there is currently no practical and useful method to simply improve the performance of the entire model. This is also a very popular research topic in the general field of machine learning. However, for these new emerging tasks in the biological field, there is still a lack of standard databases for algorithm comparison and attempts for new ideas.

Finally, we generated a series of attribution maps to visually interpret the trained model. Interpretability remains a major challenge in biological research because biological data usually have more noise than images or text. In recent years, several methods (e.g., DeepLIFT, IG, and SmoothGrad) have achieved some degree of success in this regard [32, 40, 54]. However, for deeper networks, there are still some shortcomings in terms of extracting and generating canonical motifs from the attribution maps [55]. Owing to the lack of a feasible guidebook, the basic method of judgment remains unquantifiable visual inspection.

## Conclusions

In this study, we proposed Multi-resBind as a new model utilizing multi-label learning and ResNet to infer in vivo protein-RNA binding preferences. Multi-resBind achieved high performance with respect to two evaluation metrics (AUROC and AP) on a collection of PAR-CLIP datasets (59 RBPs in total). Attribution maps revealed that the binding preferences learned from the structure or region context are biologically relevant. We hope that this model will provide a powerful tool to predict unknown binding sites in vivo and contribute toward gaining a better understanding of the underlying binding mechanisms of protein-RNA interactions.

## Supplementary Information

**Additional file 1.** Supplemental information includes Files S1–S7, Tables S1–S4, and Figs. S1–S4.

**Additional file 2: File S1.** Performance comparison of AUROC and AP scores between DeepRiPe and Multi-resBind.

**Additional file 3: File S2.** Performance comparison of AUROC scores according to different types of input data and their combinations using the Multi-resBind model. Structural profiles (PU) were calculated using RNAplfold.

**Additional file 4: File S3.** Performance comparison of AP scores using different types of input data and their combinations using the Multi-resBind model. Structural profiles (PU) were calculated using RNAplfold.

**Additional file 5: File S4.** Performance comparison of AUROC scores among different types of input data and their combinations under the Multi-resBind model. Structural profiles consisting of six categories, stem (S), hairpin (H), bulge (B), internal (I), multibranch (M), and exterior (E) loops, were calculated using CapR.

**Additional file 6: File S5.** Performance comparison of AP scores using different types of input data and their combinations under the Multi-resBind model. Structural profiles consisting of six categories, stem (S), hairpin (H), bulge (B), internal (I), multibranch (M), and exterior (E) loops, were calculated using CapR.

**Additional file 7: File S6.** Performance comparison of AUROC scores using different loss functions under the Multi-resBind model.

**Additional file 8: File S7.** Performance comparison of AP scores with different loss functions under the Multi-resBind model.

## Data Availability

The original PAR-CLIP data were obtained from a publicly available website (https://ohlerlab.mdc-berlin.de/software/DeepRiPe_140/). Two low datasets consist of RNA secondary structural profiles predicted by computation tools can be downloaded from https://doi.org/10.5281/zenodo.4743499. The two additional eCLIP dataasets (K562 and HepG2) can also be downloaded from https://doi.org/10.5281/zenodo.5508803. The results of data analysis and code for Multi-resBind are available at https://github.com/tjustorm/Multi-resBind.

## Abbreviations

AP: Average precision
AUROC: The area under the receiver operating characteristic curve
BCE: Binary cross-entropy
CLIP: Ultraviolet cross-linking and immunoprecipitation
CLIP-seq: Ultraviolet cross-linking and immunoprecipitation coupled to high-throughput sequencing
CNN: Convolutional neural network
icSHAPE: In vivo click selective 2-hydroxyl acylation and profiling experiment
IG: Integrated gradient
PAR-CLIP: Photoactivatable ribonucleoside enhanced cross-linking and immunoprecipitation
PWM: Position weight matrix
ResNet: Residual network
RBD: RNA-binding domain
RBP: RNA-binding protein
