# Supplementary material for: Multi-resBind: a residual network-based multi-label classifier for in vivo RNA binding prediction and preference visualization
Source: BMC Bioinformatics. 2021 Nov 15;22:554. doi: 10.1186/s12859-021-04430-y (PMC8594109; doi:10.1186/s12859-021-04430-y)
Supplement: Supplementary file 1 — Additional file 1. Supplemental information includes Files S1–S7, Tables S1–S4, and Figs. S1–S4. [file 12859_2021_4430_MOESM1_ESM.docx]

Supplementary Information:

Multi-resBind: a residual network-based multi-label classifier for *in vivo* RNA binding prediction and preferences visualization

Shitao Zhao^1^ and Michiaki Hamada^2,3,4^

^1^Waseda Research Institute for Science and Engineering,

Waseda University, Okubo, Shinjuku-ku, Tokyo 169-8555, Japan

^2^Department of Electrical Engineering and Bioscience, Faculty of Science and Engineering, Waseda University, Okubo, Shinjuku-ku,Tokyo 169-8555, Japan

^3^Computational Bio Big-Data Open Innovation Laboratory, National Institute of Advanced Industrial Science and Technology, Okubo,Shinjuku-ku, Tokyo 169–8555, Japan

^4^Graduate School of Medicine, Nippon Medical School, Sendagi, Bunkyo-ku, Tokyo 113–8602, Japan

Supplemental information includes Files S1–S7, Tables S1–S4, and Figures S1–S4.

File S1: Performance comparison of AUROC and AP scores between DeepRiPe and Multi-resBind.

File S2: Performance comparison of AUROC scores according to different types of input data and their combinations using the Multi-resBind model. Structural profiles (PU) were calculated using RNAplfold.

File S3: Performance comparison of AP scores using different types of input data and their combinations under the Multi-resBind model. Structural profiles (PU) were calculated using RNAplfold.

File S4: Performance comparison of AUROC scores among different types of input data and their combinations under the Multi-resBind model. Structural profiles consisting of six categories, stem (S), hairpin (H), bulge (B), internal (I), multibranch (M), and exterior (E) loops, were calculated using CapR.

File S5: Performance comparison of AP scores using different types of input data and their combinations under the Multi-resBind model. Structural profiles consisting of six categories, stem (S), hairpin (H), bulge (B), internal (I), multibranch (M), and exterior (E) loops, were calculated using CapR.

File S6: Performance comparison of AUROC scores using different loss functions under the Multi-resBind model.

File S7: Performance comparison of AP scores with different loss functions under the Multi-resBind model.

Table S1. The comparison experiments for DeepRiPe and Multi-resBind on eCLIP datasets.

| Dataset | Mean AUROC (DeepRiPe) | Mean AUROC  (Multi-resBind) | Mean AP  (DeepRiPe) | Mean AP  (Multi-resBind) |
| --- | --- | --- | --- | --- |
| K562_low | 0.7943 | **0.7965** | **0.3330** | 0.3236 |
| K562_med1 | 0.8310 | **0.8414** | 0.3878 | **0.3995** |
| K562_med2 | 0.8203 | **0.8329** | 0.3756 | **0.3935** |
| K562_high1 | 0.8731 | **0.8764** | 0.5539 | **0.5630** |
| K562_high2 | 0.8256 | **0.8421** | 0.4789 | **0.5082** |
| HepG2_low | 0.7833 | **0.7920** | 0.2930 | **0.2980** |
| HepG2_med1 | 0.8810 | **0.8890** | 0.5147 | **0.5286** |
| HepG2_med2 | 0.8024 | **0.8141** | 0.3740 | **0.3898** |
| HepG2_high1 | 0.9170 | **0.9252** | 0.7496 | **0.7682** |
| HepG2_high2 | 0.8754 | **0.8926** | 0.5342 | **0.5797** |

*Notes*: The numbers marked in bold represent the maximum value under the respective eCLIP dataset using the same valuation metric. K562_low,

K562_med1, K562_med2, K562_high1 and K562_high2 represent five categories with different peaks in eCLIP datasets of cell line K562. HepG2_low, HepG2_med1, HepG2_med2, HepG2_high1 and HepG2_high2 represent five categories with different peaks in eCLIP datasets of cell line HepG2.

Abbreviations: AUROC, area under the receiver operating characteristic curve; AP, average precision;

Table S2. The ablation experiments for residual network block and pooling layer

| Network architecture | Mean AUROC (low) | Mean AUROC (med) | Mean AUROC (high) | Mean AP (low) | Mean AP (med) | Mean AP (high) |
| --- | --- | --- | --- | --- | --- | --- |
| CNN 1 layer + max pooling | 0.8409 | 0.8720 | 0.8285 | 0.2511 | 0.4337 | 0.4194 |
| CNN 1 layer + average pooling | 0.8666 | 0.8719 | 0.8295 | 0.3009 | 0.4333 | 0.4207 |
| CNN 1 layer + Resnet block + max pooling | 0.8911 | 0.8878 | 0.8406 | 0.3584 | 0.4790 | 0.4467 |
| CNN 1 layer + Resnet block + average pooling | **0.8976** | **0.8889** | **0.8417** | **0.3808** | **0.4826** | **0.4486** |

*Notes*: The numbers marked in bold represent the maximum value of the same dataset under the respective evaluation metric. The low, med and high represent three categories with different peaks in PAR-CLIP datasets.

Abbreviations: AUROC, area under the receiver operating characteristic curve; AP, average precision;

Table S3. Performance of Multi-resBind model with different types of input data.

| Data types | Data dimensions | Mean AUROC | Mean AP |
| --- | --- | --- | --- |
| Sequence | (150,4) | 0.8809 | 0.3372 |
| Structure | (150,6) | 0.6466 | 0.0969 |
| Region | (150,4) | 0.6710 | 0.0912 |
| Sequence and structure | (150,10) | 0.8832 | 0.3427 |
| Sequence and region | (150,8) | **0.8976** | **0.3808** |
| Region and structure | (150,10) | 0.7715 | 0.1710 |
| Sequence, structure, and region | (150,14) | 0.8966 | 0.3720 |

*Notes*: The numbers marked in bold represent the maximum value under the respective evaluation metric. Structural profiles consisting of six categories, stem (S), hairpin (H), bulge (B), internal–(I), multibranch (M), and exterior (E) loops, were calculated using CapR.

Abbreviations: AUROC, area under the receiver operating characteristic curve; AP, average precision.

Table S4. Performance comparison of different loss functions.

| Loss function | Mean AUROC | Mean AP |
| --- | --- | --- |
| Class_aware | 0.8966 | 0.3720 |
| BCE | 0.8981 | 0.3763 |
| WBCE | 0.8934 | 0.3523 |
| Focal loss | **0.8993** | 0.3745 |
| ASL | 0.8975 | **0.3765** |

*Notes*: The numbers marked in bold represent the maximum value under the respective evaluation metric.

Abbreviations: AUROC, area under the receiver operating characteristic curve; AP, average precision; BCE, binary cross-entropy; WBCE, weighted binary cross-entropy; ASL, asymmetric loss.

Figure S1: Violin plot (a) and box plot (b) of Multi-resBind model performance using different types of input data: sequence (seq), structure (struc), region, and their combinations. AUROC, area under the receiver operating characteristic curve.

(a)

(b)

Figure S2: Count distribution of the positive labels among RNA-binding proteins (RBPs).

Figure S3: Relationship between the performance of DeepRiPe and the number of positive labels in each class for (a) area under the receiver operating characteristic curve (AUROC) and (b) average precision (AP). RBP: RNA-binding protein.

(a)

(b)

Figure S4: Contribution maps for model interpretation using a zero vector as the input. Contribution maps for model interpretation were performed using the integrated gradients method, in which the reference point was a zero vector. The sequence logos corresponding to attribution maps were generated through the RCAS profiles of the RNA-binding protein binding sites.
